# Evaluation of the Lower Punctum Parameters and Morphology Using Spectral Domain Anterior Segment Optical Coherence Tomography

**DOI:** 10.1155/2015/591845

**Published:** 2015-05-21

**Authors:** Riham S. H. M. Allam, Rania A. Ahmed

**Affiliations:** Ophthalmology Department, Kasr El Aini School of Medicine, Cairo University, Cairo, Egypt

## Abstract

*Purpose*. To study features of the lower punctum in normal subjects using spectral domain anterior segment optical coherence tomography (SD AS-OCT).* Methods*. Observational cross-sectional study that included 147 punctae (76 subjects). Punctae were evaluated clinically for appearance, position, and size. AS-OCT was used to evaluate the punctal shape, contents, and junction with the vertical canaliculus. Inner and outer diameters as well as depth were measured.* Results*. 24 males and 52 females (mean age 44 ± 14.35 y) were included. Lower punctum was perceived by OCT to be an area with an outer diameter (mean 412.16 ± 163 *μ*m), inner diameter (mean 233.67 ± 138.73 *μ*m), and depth (mean 251.7 ± 126.58 *μ*m). The OCT measured outer punctum diameter was significantly less than that measured clinically (*P*: 0.000). Seven major shapes were identified. The junction with the vertical canaliculus was detectable in 44%. Fluid was detected in 34%, one of which had an air bubble; however, 63% of punctae showed no contents and 4% had debris.* Conclusions*. AS-OCT can be a useful tool in understanding the anatomy of the punctum and distal lacrimal system as well as tear drainage physiology. Measuring the punctum size may play a role in plugs fitting.

## 1. Introduction

The punctum is the only visible part of the lacrimal drainage system. It is classically defined as the opening situated on top of the lacrimal papilla present at the medial aspect of the upper and lower lid margins. The lower punctum is placed slightly lateral to the upper. Each punctum connects to a 2 mm vertical canaliculus that turns medially for 8 mm to finally open in the lacrimal sac either separately or after joining the other canaliculus forming a common canaliculus. Both punctae are normally directed towards the globe; hence the lid should be everted to allow their examination [1].

Understanding the punctum anatomy is important for better evaluation of its disorders causing epiphora, mainly stenosis or total occlusion [2]. Punctal anatomy could be important for designing and fitting plugs as well as inserting other nasolacrimal stents [3]. It could also play a role in understanding the physiology of tear drainage.

Few studies have been concerned with studying the punctum anatomy and imaging. Wawrzynski et al. have recently described the possibility of using anterior segment optical coherence tomography (AS-OCT) to provide high-resolution images of the punctum and proximal canaliculus with promising results [4].

This is a noninvasive noncontact imaging technique that uses infrared light to study ocular structures in vivo. It has been a useful tool that provides high-resolution tomographic cross-sectional imaging of the anterior segment since its first production by Izatt et al. in 1994 [5].

In the current work, AS-OCT was used to study in vivo anatomical features of the lower punctum in asymptomatic Egyptian subjects.

## 2. Patients and Methods

This is an observational cross-sectional study that took place from May 2014 to August 2014. Healthy asymptomatic volunteers, 20–60 years old, were recruited from the outpatient service at Kasr El Aini School of Medicine. Data collection conformed to all local laws and was compliant with the principles of the Declaration of Helsinki.

A detailed history was taken for chronic use of topical eye drops or previous eye surgery. All subjects were clinically evaluated using the slit lamp by one experienced oculoplastic surgeon (Rania A. Ahmed). All were assessed for lid and lid margin position, snap back test, lid distraction test, punctual visibility and relation to the globe, the tear meniscus height, and the position of the lacrimal papilla in relation to the lid margin whether normal, pouting, or displaced. Ocular surface examination including corneal staining and break-up time (BUT) was performed to detect ocular dryness.

Patients with history of chronic topical antiglaucoma drops or previous ocular or lacrimal surgeries were excluded. We also excluded patients with high tear meniscus that is >0.2 mm, positive regurge, lid laxity, lid margin malpositions (such as ectropion, entropion, or lid retraction), medial lid masses obscuring the punctum, and dryness where BUT was <5 seconds as well as clinically invisible punctae due to stenosis or membranes.

The punctal diameter was then clinically evaluated by the largest gauged blunt cannula that could be inserted through the punctal opening without dilatation under topical anesthesia. The measured gauge was converted into microns of its outer diameter using conversion tables for correlation and comparison.

All patients were then evaluated by a single experienced physician (Riham S. H. M. Allam) utilizing SD AS-OCT, RTVue model-RT100 CAM system (Optovue Inc., Fremont, CA, USA) version 6.2 with the use of the cornea/anterior module-short lens (CAM-S) of 10 mm working distance. The lower lid punctum was exposed by gently everting the medial part of the eyelid without pressure or stretching, thus bringing the vertical canaliculus into the axial plane.

**Figure 1:**
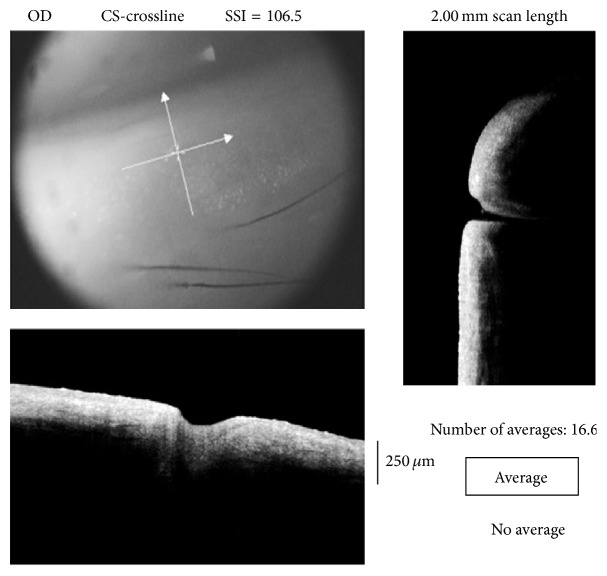
Image acquisition using crossline centered at the lower punctum (upper left); the vertical line scan explores the depth of the punctum and scans the junction between the punctum and vertical canaliculus (right) while the horizontal line scan demonstrates the extent of the punctum and can measure the outer diameter (bottom left).

A crossline scan (2 mm × 2 mm) was centered on the studied punctum for alignment; then a cross-scan was obtained as shown in Figure 1. For better imaging, external illumination was adjusted using the two short gooseneck cables with red LED. Three scans were obtained for each examined eye. The operator then chose the images with the clearest, widest, and deepest measurable diameters.

**Figure 2:**
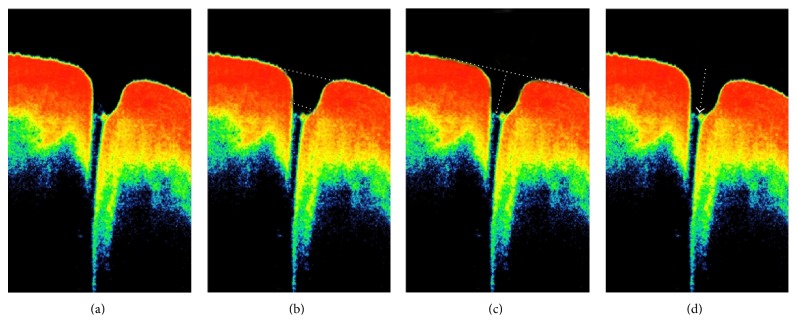
A normal punctum as shown by AS-OCT. (a) Evaluation of shape and contents of the punctal area. (b) Measurement of outer and inner punctal diameters. (c) Measuring the punctal height by drawing a line tangent to the lower lid margin and passing across the punctum, the distance measurement tool is then placed and a perpendicular line is drawn from the tangent line towards the floor of the punctum. (d) Evaluation of the junction between the punctum and the vertical canaliculus (arrow).

For all the studied subjects, the overall punctal shape and contents were evaluated (Figure 2(a)); then the outer, that is, towards the lid margin, and the inner, that is, towards the canaliculus, diameters were measured using the adjustable distance measurement tool (Figure 2(b)). The punctal depth was determined by drawing a line tangential to the lower lid margin across the punctal outer opening; then a perpendicular line was drawn towards the base of the punctum (Figure 2(c)). Medial tear meniscus height (TMH) and tear meniscus area (TMA) were also measured.

The junction of the punctum with the vertical canaliculus was identified through detecting the sudden narrowing in the lumen diameter and/or the morphological thinning of the lining epithelium. The abrupt narrowing of the lumen was followed as shown in Figure 2(d) into the beginning of the vertical canaliculus. We could interpret this as the junction between the punctum and the vertical canaliculus. The internal punctal diameter was taken as the horizontal line drawn just above this junction as in Figure 2(b).

Descriptive terms to the overall shape of the section were suggested while considering the relation between inner and outer diameters and depth as well as the angulation of the medial and lateral walls towards the inner punctum. A questionnaire was designed and forty doctors (ophthalmologists and nonophthalmologists) were asked to give a description for each image. Descriptions that obtained more than 65% agreement were used to describe the morphological appearance of the punctum.

Data was analyzed using SPSS V.16 statistical software for Windows (SPSS Inc., Chicago, IL, USA). Descriptive statistics were calculated and numerical data were summarized as mean ± SD while categorical data were summarized in percentages. Paired *t*-test was used to compare numerical data between right and left eyes. Correlation between ordinal variables was done using Mann-Whitney *U* test, Chi square, Pearson Chi square, and correlation.

## 3. Results

This study included 147 eyes of 76 volunteers: 24 males and 52 females with mean age of 44 ± 14.35 years. On OCT assessment, the lower punctum was found to be an area with mean outer diameter 412.16 ± 163 *μ*m, mean inner diameter 233.67 ± 138.73 *μ*m, and mean depth 251.7 ± 126.58 *μ*m.

Clinical punctal size and parameters measured by the AS-OCT in each eye are summarized in Table 1. There was no statistically significant difference between the Rt and Lt eyes parameters.

**Figure 3:**
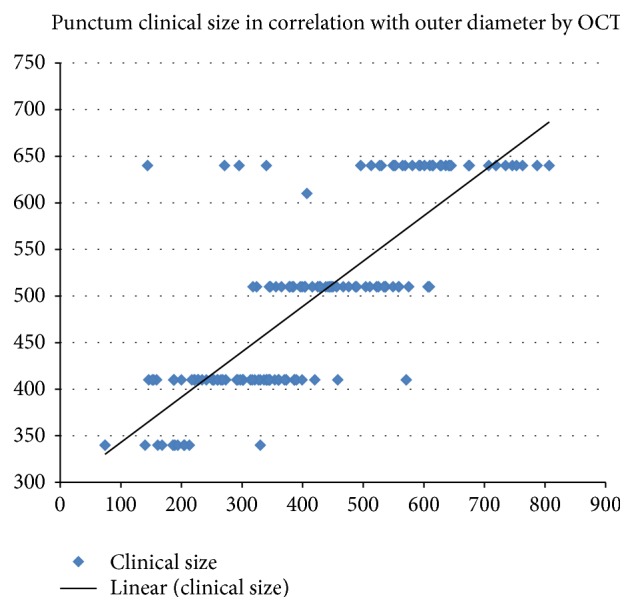
Correlation between the OCT measured outer diameter and the clinically measured punctal size showing a positive linear correlation.

A strong positive linear correlation (Figure 3) was found between the clinical measurement of the punctum size using the blunt cannula and the outer diameter measured by the OCT (*R*: 0.795). However, the readings obtained by the OCT were significantly less than the clinical measurements (*P*: 0.000).

Another positive linear correlation was found between the overall OCT measured inner punctum diameter and outer punctum diameter whether measured by OCT or clinically (*R*: 0.688 and 0.615, resp.). Both correlations were statistically significant (*P*: 0.000).

On the other hand, overall TMH and TMA showed a weak negative correlation with the outer punctual diameter measured clinically and by OCT as well as the inner punctal diameter measured by OCT. None of these correlations were statistically significant (*P* > 0.05). Values are summarized in Table 2.

The junction between the punctum and the vertical part of the lower canaliculus could be detected in 64 eyes (44%). No contents were detected within the scanned punctual area in 93 eyes (63%), yet it contained fluid in 50 eyes (34%) which was clear in 15 eyes (10%) and turbid in 35 eyes (24%) of the whole sample. An air bubble was found in one of the cases with clear fluid. Debris with no fluid was detected in 4 eyes (3%).

**Figure 4:**
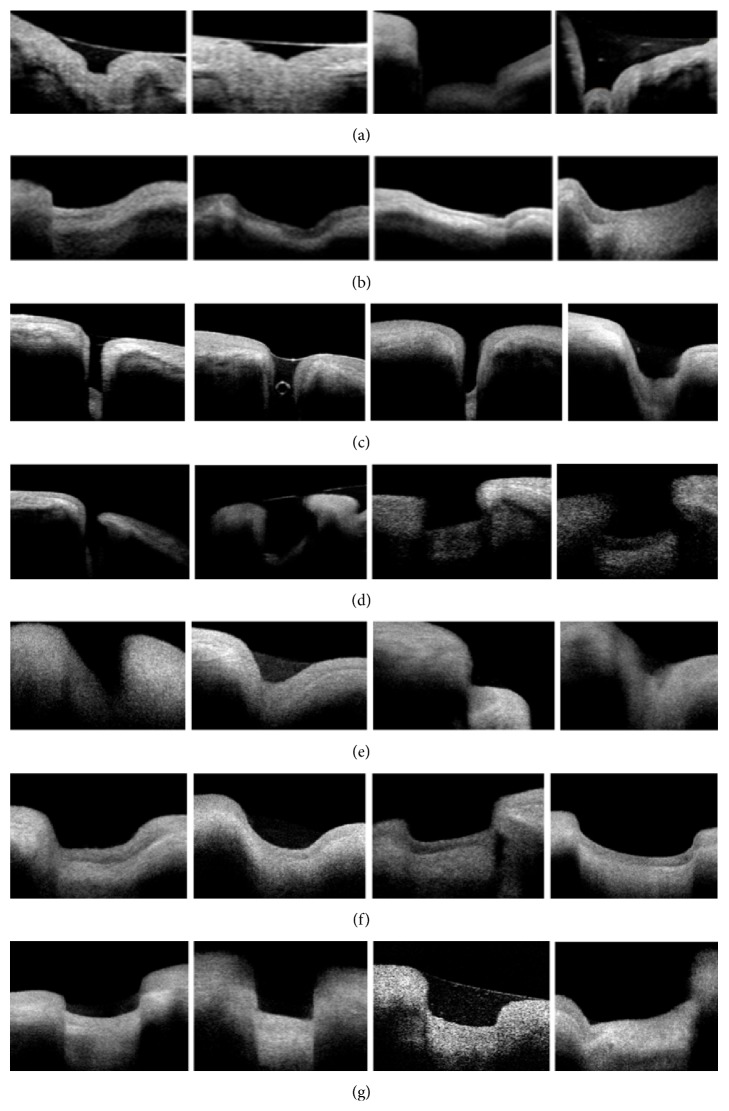
Punctal morphologies as seen by OCT: (a) bicornuate, (b) creeping edge, (c) deep (an air bubble is also detected), (d) flask shaped, (e) funnel shaped, (f) saucer shaped and (g) square shaped.

Seven major morphological types (Figure 4) were detected and classified according to the overall shapes, relation between inner and outer diameters, depth, and the angulation of the medial and lateral walls towards the inner punctum. The criteria and numbers of each detected type in this sample are summarized in Table 3.

## 4. Discussion

Anterior segment OCT (AS-OCT) is an established noncontact imaging modality that uses infrared light of 840 nm wavelength to provide informative structural images of the anterior segment. Wawrzynski et al. have recently proven its feasibility in the assessment of the lower punctum and the related vertical canaliculus [4].

The punctum has always been subjectively evaluated; however, consistency does not imply accuracy especially with absence of absolute standards. Kashkouli et al. suggested a grading system for punctal stenosis [2] based on slit lamp examination and its validity was further confirmed considering the interobserver variation [6].

In vivo imaging modalities of the punctum and canalicular system are limited. Ultrasound biomicroscopy has been studied to evaluate these structures; however, it provides limited data with lower resolution images. Additionally, it requires contact of the probe with the tissues rendering it uncomfortable for the patient [7]. AS-OCT, on the other hand, can provide higher resolution images while keeping the patient more comfortable [4].

In the current study, the right and left sides showed no difference in all the measured parameters. The OCT measurement of the outer diameter of the punctum had a direct strong correlation with the clinical measurement, yet it showed significantly smaller values. The higher readings obtained clinically are possibly due to the ability of the punctum to stretch in order to accommodate the inserted blunt cannula. The OCT measurements were less than the reported values by Timlin et al. [8] (mean 615 ± 367 *μ*m) and more than the recently published work of Wawrzynski et al. (mean 247 ± 78 *μ*m) [4].

An evident change in the punctum configuration and changes in the epithelium thickness were found and documented. The junction between the punctum and vertical canaliculus was identified in 44% of the examined eyes. Hence, the term of inner diameter was suggested. It ranged from 41 to 796 *μ*m and was higher than values reported by Timlin et al. in their exploratory study (0–99 *μ*m) [8]. The inner diameter measurements showed direct strong correlation with the outer diameter measured clinically and by AS-OCT.

The depth measured in our study ranged from 82 to 883 *μ*m (mean 251.7 ± 126.58 *μ*m) compared to Timlin et al. who reported the maximum detected depth to be 500 [8]. These values are considered very short compared to Wawrzynski et al. who reported depth ranging from 392 to 1242 *μ*m (mean of 753 ± 216 *μ*m) and presumed it to be the vertical canaliculus length [4]. They also found that it was significantly shorter than the anatomically stated length of 2 mm. The cadaveric studies are few where some stated that the vertical canaliculus is around 1 mm while others reported length of 2.82 mm [9, 10].

We considered the depth measured in the current study to represent punctal rather than vertical canaliculus depth. This was based on detecting the inner punctum area, variable shapes of punctae, and the recorded readings.

This differs from a study by Kamal et al., which used the same machine (RTVue) to measure three dimensions: the mean maximum punctual diameter, mid-canalicular diameter, and vertical canalicular height. The study considered the photographed area as the punctum and vertical canaliculus altogether and only took one measurement as a representative of the punctal dimensions without considering the inner punctum as a surrogate measurement. This might explain their higher mean vertical canalicular length. However, their 3D and En face images gave a topographic representation of the proximal lacrimal system which makes OCT a very desirable imaging modality for the surface topography of the punctum [11].

Inability to detect the punctal junction with the canaliculus in 56% of our cases could be due to limited lid tissues penetration by infrared light and the possible collapse of the canaliculus. Kakizaki et al., using cadaveric dissection, had found that the vertical canaliculus of the lower lid is usually laterally inclined by 5 degrees, which could contribute to the inability to detect such junction by OCT imaging [12].

The large sample size in the current study is one of the factors that could explain the differences in the measured punctal parameters as compared to the other studies by Wawrzynski et al. (36 punctae) and Timlin et al. (22 punctae) [4, 8]. The different OCT machines used and the examination protocols are contributing factors.

There was no difference between the right and left sides as regards the measured parameters. The reported TMH and TMA were consistent with the values of medial tear meniscus reported in normal subjects studied by Park et al. using spectral domain OCT [13].

However, there was a weak negative correlation between TMH and TMA, respectively, with the clinical and OCT measured punctal diameters that were statistically insignificant. This reflects the incorporation of multiple physiological mechanisms in tear drainage and tear film maintenance and is in accordance with the studies that showed that the lower punctum is not the major route for tear elimination [14, 15].

In conclusion, the use of AS-OCT in assessing the punctum and proximal lacrimal system in vivo opens new venues for understanding the anatomy and the physiology of the lacrimal system. It can be also used to track punctal changes related to chronic topical medications as well as changes following lacrimal or ocular surgeries and ocular surface disorders. The different morphological shapes could be of value in punctal plug fitting.

Although examining what lies beneath is an exciting idea, the inability of examining the upper punctum is one of the limitations facing this modality. In addition, imaging could be long and bothersome to the patient especially at the start of the examiner's learning curve.

Further structured studies with larger numbers are required to establish a morphological scoring system and to correlate the shapes with lacrimal tear film as well as the patient's symptoms.

## Figures and Tables

**Table 1:** Punctal and tear meniscus parameters in both eyes of the examined group.

Parameter	Rt		Lt		*P* value
	Mean (um)	±SD	Mean (um)	±SD	
Clinical measurement	496.45	105.207	492.68	93.945	0.365
Outer diameter	424.65	170.546	404.25	159.139	0.416
Inner diameter	234.35	144.375	238.80	134.416	0.819
Depth	250.44	109.880	249.56	142.599	0.965
TMH	286.13	176.631	295.90	194.949	0.091
TMA (mm^2^)	0.04201	0.056719	0.05393	0.181726	0.070

**Table 2:** Pearson correlation and *P* value for overall TMH and TMA correlated with measured punctal diameters.

	TMH		TMA	
	*R*	*P* value	*R*	*P* value
Outer diameter OCT	−0.129	0.386	−0.157	0.288
Outer diameter clinically	−0.161	0.232	−0.199	0.136
Inner diameter OCT	−0.112	0.137	−0.115	0.264

**Table 3:** Characteristics of the major morphological punctal types detected by OCT and their frequency.

Category	Punctal depth	Outer : inner diameter correlation	Angle of medial wall to inner punctum	Angle of lateral wall to inner punctum	Number (%)
Bicornuate^∗^	Deep	2 : 1	Right/obtuse	Right/obtuse	8 (5.4%)
Creeping edge	Shallow	2 : 1	Right	Obtuse	38 (25.8%)
Deep	Deep	1 : 1	Right/parallel	Right/parallel	13 (8.8%)
Flask-shaped^∗∗^	Deep	1 : 1.4	Acute	Acute	7 (4.8%)
Funnel-shaped^∗∗∗^	Shallow	3 : 1	Obtuse	Obtuse	27 (18.4%)
Saucer-shaped	Shallow	2 : 1	Obtuse	Obtuse	38 (25.8%)
Square-shaped	Shallow	1 : 1	Right/parallel	Right/parallel	16 (10.8%)

^∗^There is a characteristic bulge in the inner punctum forming with the walls. A W-shaped appearance. ^∗∗^The two diverging walls join the inner punctum in a rounded junction. ^∗∗∗^The two walls form a V-shaped punctum.
